# Brief intervention to promote smoking cessation and improve glycemic control in smokers with type 2 diabetes: a randomized controlled trial

**DOI:** 10.1038/srep45902

**Published:** 2017-04-05

**Authors:** William H. C. Li, M. P. Wang, T. H. LAM, Yannes T. Y. Cheung, Derek Y. T. Cheung, Y. N. Suen, K. Y. Ho, Kathryn C. B. Tan, Sophia S. C. CHAN

**Affiliations:** 1 School of Nursing, The University of Hong Kong, HKSAR; 2 School of Public Health, The University of Hong Kong, HKSAR; 3 Department of Medicine, The University of Hong Kong, HKSAR.

## Abstract

The aim of the study was to examine the effects of a brief stage-matched smoking cessation intervention group compared with a control group (with usual care) in type 2 diabetes mellitus patients who smoked by randomized controlled trial. There were 557 patients, randomized either into the intervention group (n = 283) who received brief (20- minute) individualized face-to-face counseling by trained nurses and a diabetes mellitus-specific leaflet, or a control group (n = 274) who received standard care. Patient follow-ups were at 1 week, 1 month, 3 months, 6 months, and 12 months via telephone, and assessment of smoking status from 2012 to 2014. Patients smoked an average of 14 cigarettes per day for more than 37 years, and more than 70% were in the precontemplation stage of quitting. The primary outcome showed that both the intervention and control groups had similar 7-day point-prevalence smoking abstinence (9.2% vs. 13.9%; p = 0.08). The secondary outcome showed that HbA1c levels with 7.95% [63 mmol/mol] vs. 8.05% [64 mmol/mol], p = 0.49 at 12 months, respectively. There was no evidence for effectiveness in promoting the brief stage-matched smoking cessation or improving glycemic control in smokers with type 2 diabetes mellitus, particularly those in the pre-contemplation stage.

Diabetes mellitus (DM) is emerging as a global epidemic. By 2030, the number of patients with DM is projected to increase to 552 million worldwide, with one-fourth in China^1^. Type 2 DM constitutes more than 90% of DM cases among Hong Kong Chinese patients and is the leading chronic non-communicable disease and cause of death^2^. Around 10.4% of DM patients smoked in 2001^3^. In 2012, male DM patients had a higher smoking rate (27%) than in the complete male population (19.1%)^4^. Growing evidence suggests that smoking has a substantial effect on morbidity and mortality rates in patients with DM^5^. The most common risks for DM patients who smoke are an increased glycated hemoglobin level (HbA1c)^6^, an increased incidence of hypoglycemia, and a greater risk of coronary heart disease, stroke, and diabetic nephropathy^7^,^8^,^9^. Smoking also increases the risk of peripheral vascular disease and resultant amputation in DM patients^10^. Given the hazardous effects of continued smoking in patients with DM, it is of paramount importance to help this vulnerable group to quit smoking. Nevertheless, intensive glycemic control and cardiovascular risk reduction are the primary objectives of treatment in patients with type 2 DM, and only recently has the American Diabetes Association recommended smoking cessation intervention as a part of standard medical care^11^.

Medical attention for smokers with DM in outpatient clinics can serve as a site for a “teachable moment” because it provides opportunities for patients to initiate smoking cessation to improve their health. It also presents health care professionals with opportunity to advise smokers to quit while they are waiting for medical consultation or follow-up^12^. Nevertheless, cigarette smoking is addictive, and quitting is very difficult and has a high rate of relapse, particularly among chronic smokers with high nicotine dependency^12^,^13^,^14^,^15^. There are smoking cessation services and guidelines provided by non-governmental organizations and hospitals locally^16^,^17^,^18^. These services are the major resources for smokers to seek cessation assistance and counselling.

The results of a recent qualitative study^19^ of smoking behavior, perceptions about quitting smoking, and factors associated with the intention to quit in Hong Kong Chinese patients with type 2 DM revealed that many had misconceptions about the association between DM and smoking. They did not realize the risks of continued smoking in relation to treatment efficacy and increased DM complications. In addition, patients claimed that they were satisfied with their present health status and that they saw no need to quit smoking. Some smokers even believed that quitting would harm their physical well-being because their bodies had become desensitized to the chemicals in tobacco after long-term smoking. While stage-matched smoking cessation intervention is a commonly used approach^20^,^21^,^22^, we found only one small randomized controlled trial (RCT) that showed an increase in the quit rate with 14.7% more than the usual care group after a stage-matched smoking cessation intervention in DM patients (95% confidence interval [CI] 8.2–21.3%, n = 280)^23^. The small sample sizes of fewer than100 used in previous studies^24^,^25^ limit the generalizability of their findings. It is imperative that there is rigorous empirical research on the effectiveness of stage-matched smoking cessation intervention. Our previous studies in outpatient clinics have revealed that patients are too impatient to undergo long-term or time-consuming intervention^26^. Cancer patients in our trial were reluctant to participate for fear that their medical consultation or procedures may be missed or delayed. In addition, study of DM patients showed, insufficient evidence for the efficacy of more intensive smoking cessation interventions such as providing a more focused counseling or interview by medical advisors through face-to-face or by telephone^27^.

Previous studies have found that smokers in Hong Kong were not ready to quit. In a trial with 1154 adult participants who were not willing to quit, the 6-month smoking abstinence rate of unmotivated patients who received the usual care and self-help smoking cessation material was 10.2%^28^. Another trial with a group of Chinese cardiac patient smokers (n = 1860) also showed that patients undergoing intervention with stage-matched counseling failed to significantly promote their quit rate compared with the control group who received placebo treatments (healthy diet brief advice)^29^. There was no significant difference in self-reported 7-day (*p* = 0.60) and 30-day (*p* = 0.55) abstinence between intervention and control groups^29^.

This study aimed to test the effectiveness of brief, stage-matched smoking cessation counseling using large sample sizes to promote smoking cessation and to reduce the health risks of smokers with type 2 DM. We hypothesized that participants in the interventional group would (a) report higher quit rates measured by self-report and biochemical validation, (b) reduce smoking cigarette consumption by at least 50%; (c) report lower HbA1c levels than those in the control group at 12 months.

## Methods

### Ethical Review

This study was registered on the Clinical Controlled Trials as ISRCTN34551140 (March 5, 2012) and approved by the Institutional Review Board of the University of Hong Kong and Hospital Authority Hong Kong West Cluster (April 18, 2008; Reference: UW08–142).

Written informed consent was obtained from all eligible participants. To ensure the rights of all participants were protected, the researchers strictly adhered to the Declaration of Helsinki and the ethical principles in designing and conducting clinical research.

### Study Participants

Patients who had medical follow-up visits at nine major hospital diabetes clinics and met the inclusion criteria were invited to participate. The inclusion criteria for the patients were (1) diagnosis of type 2 DM for at least 6 months, (2) age of 18 years or above, (3) smoking at least two cigarettes per day over the past 30 days, and (4) able to communicate in Cantonese (around 90% of the population’s language is Cantonese stated by the Census report in 2014^30^). The reason for targeting patients diagnosed with type 2 DM for at least 6 months was that their clinical condition should be more stable. Patients were excluded if they (1) were clinically too ill, (2) failed to have verbal response or poor cognitive state, (3) were unable to complete the questionnaires, and (4) were engaged in other smoking cessation programs.

### Sample size

The computer programme G*Power was used to calculate the sample size^31^. According to the results of our previous study, the quit rate for smokers who were not willing to quit smoking was 10.2%^28^. To detect a rate difference of 8% (i.e., an odds ratio (OR) of about 2 for quitting smoking) in the intervention group, 322 patients were needed in each group to achieve statistical power of 80% (commonly used in smoking cessation trials^32^,^33^,^34^) at a 5% significance level of a two-sided chi-squared test. Assuming a 15% attrition rate over the 6-month study period^35^, 379 patients were required in each group to achieve a significant outcome. Hence, the total sample size should be 758. Assuming equal standard deviations of 1.3 in either group, the large sample size achieved sufficient statistical power (>0.99) of an independent-samples *t*-test to detect a difference of 0.5% in the mean HbA1c level between the intervention group and the control group with a significance level of 5%.

### Theoretical framework

Smokers may tend to reject smoking cessation intervention when they are not ready to quit^36^,^37^. Therefore, the use of the Stages of Change Model^38^, a well-known protocol for smoking cessation with a short 20 min intervention time can quickly access the stage of readiness of the patient and provide tailored smoking cessation strategies. Motivational intervention was effective in enhancing cessation rate versus control showed in a meta-analysis review on 31 trials from adolescents to adults (OR, 1.45; 95% CI: 1.14–1.83)^39^.

The transtheoretical model of behavioral change (TTM)^38^ was used to guide the development of the targeted smoking cessation intervention for smokers. It is a well-known framework in psychotherapy for behavioral changes and has been validated for more than 20 years^40^. The core constructs of the TTM include the stage of change, the process of change, the pros and cons of changing, and self-efficacy. Prochaska and DiClementte^41^ identified various stages and processes of self-change in smoking that correspond to the five stages of change in the TTM: pre- contemplation; (2) contemplation; (3) preparation stage; (4) action stage; and (5) maintenance. Prochaska and DiClementte^41^ pointed out that individuals differ in each stage of readiness to change their behavior and that interventions should therefore be tailored according to the individual’s stage of change.

### Interventions

The patients in the intervention group received a 20-min face-to-face individualized counseling session by nurse counselors who were specifically trained for the DM smoking cessation counseling. Due to the short intervention time, we defined it as a brief intervention compared with the standard protocol for telephone smoking cessation counseling of 35 to 45 min per session^42^. Studies have shown that a brief intervention such as oral advice or brief counseling would be cost-effective and easy to implement for physicians or nurses in clinic or hospital settings to promote the rate of quitting in patients^43^,^44^. Another intervention study was a subject-centered motivational intervention that focused on promoting the reasons to quit, on emphasizing this choice, on achieving goal and on the subject’s resistance to quitting^45^. This intervention helped to build up motivations and create a comfortable atmosphere to communicate with the patients for behavioral counseling. It was focused on stage-matched counseling for smoking cessation that was adopted from a widely used local protocol^46^ based on the clinical practice guidelines using the “Five A’s” approach, which refers to (1) asking about tobacco use; (2) advising quitting; (3) assessing the subject’s willingness to quit; (4) assisting in the attempt to quit; and (5) arranging follow-up. The approach was strongly evidence-based and confirmed the benefit of smoking cessation treatment in the health-care system^47^.

Patients in our study also received a DM-specific leaflet and a self-help pamphlet on quitting smoking. The leaflet summarized the relationships between smoking and diabetic complications, HbA1c levels and the common misconceptions about quitting. These relationships were emphasized in the intervention groups as a trigger for considering quitting. Specifically, the items stressed were smoking-related diseases, including coronary heart disease, peripheral vascular disease, cataracts, erectile dysfunction (for male patients), and other complications of DM. Thus, the intervention provided patients with the risk information of smoking towards health. Previously, it has been shown that health information with comprehensive context motivates persons to consider the health issues and enhance the perceptions of health risk^48^. At the 1-week and 1-month follow-up visits, in addition to the collection of the smoking status information, the patients received a booster of brief intervention aimed at enhancing their self-efficacy and overcoming their barriers to quitting. The complete follow-up visits were about 30 min, which was similar to the baseline intervention. To ensure the nurse counsellors adhered to the intervention protocol, we periodically checked the intervention process.

The control group received the usual care that focused on glucose-oriented diabetic control provided by DM clinics. These patients also received simple, brief advice and a self-help pamphlet on quitting smoking. In 2010, there were 11240 hotline-counseling sessions for smoking cessation completed by the Hong Kong Hospital Authority (HA), and 43% of the 4156 clients who attended these clinics ceased smoking^49^. It is challenging for smokers to quit if no interventions are strategically provided to address their health issues due to the lack of confidence and sustainability in implementation^36^,^37^. The HA also provides a usual care guideline for DM patients, which briefly mentions contacts and referral information for smoking cessation services^50^. Previous studies have shown that DM smokers are more likely to be in the pre-contemplation stage of quitting and have less interest in their health, which suggests that they may more likely reject smoking cessation intervention^51^.

### Study Design

The RCT was used to examine the effectiveness of the brief intervention to promote smoking cessation and improve glycemic control in smokers with type 2 DM. An exhaled carbon monoxide test (≥4 ppm) was used to confirm smoking status before randomization.

The nurse counselors assigned patients to the intervention or control group individually by simple randomization according to serially numbered sealed opaque envelopes containing a random number generated by the computer for each study site. The nurse counselors were unaware of the random sequence, which another researcher generated.

### Study Assessments and Outcomes

The primary outcome was the self-reported 7-day point-prevalence smoking abstinence at 12 months. The secondary outcomes were (a) the HbA1c level at 12 months, (b) the validated 12-month quit rate, (c) a reduction in smoking by at least 50% at 12 months compared with baseline, (d) changes in patients’ stage of readiness to quit at 12 months, and (e) the number of quit attempts over the past 12 months.

### Demographics and Smoking Characteristics

Baseline data included smoking history, demographic, socioeconomic, and clinical characteristics obtained from each subject using a structured questionnaire administered by a trained nurse counselor. The content of the structured questionnaire included smoking-related information such as daily cigarette consumption, nicotine dependency as assessed by the Fagerstrom test, the stage of readiness to quit according to TTM, and previous quit attempts. The HbA1c levels of patients at baseline and during the follow-ups were obtained from their medical records.

### Data Collection

The data collection period including recruitment and follow-up were between March 2012 and April 2014.The nurse counselors conducted follow-up interviews by telephone at 1 week and 1 month after the initial contact. Three more follow-up telephone contacts at 3, 6, and 12 months were conducted by another nurse counselor who was blinded to the group assignment. The counselor collected information on smoking status and self-efficacy^52^ to refrain from smoking. Information on the medical history of hospitalization in the past 3 months and the HbA1c levels was also obtained from the respective hospitals at 3, 6, and 12 months. Patients who reported that they had successfully quit after 6 or 12 months or had reduced cigarette consumption by at least 50% at 6 months were invited to test on saliva cotinine (<115 ng/mL NicAlert strips (www.nymox.com)^53^ and exhaled carbon monoxide (<4 ppm to confirm quitting or <9 ppm for smoking reduction)^54^. Participants received an incentive offer of HK$300 (US$39) per subject to cover time cost.

### Statistical Analysis

All data were analyzed using SPSS 23.0 standard version for Windows. For dichotomous outcomes, a chi-squared or Fisher’s exact test was used to examine the differences between the intervention and control groups. The adjusted (for step-up adjustment of anti-diabetes medication) mean difference in the HbA1c levels at 12 months between groups was tested with the generalized linear model. An independent-samples t-test was used to examine the differences in the HbA1c levels between quitters and non-quitters at baseline and 12 months. A Generalized Estimating Equation (GEE) analysis was conducted to examine changes in the HbA1c levels in quitters and non-quitters from baseline to 12 months adjusted to time, grouping of intervention and usual care, age and gender. Cessation at 6 and 12 months was compared by unadjusted odds ratios (ORs) using univariable logistic regression. We used an intention-to-treat analysis^55^. In particular, multiple imputation was used to compute missing data for all outcome variables, except quitting and smoking reduction. For these two variables, a conservative approach was adopted, in which participants who dropped out were considered to be unable to quit or reduce their cigarette consumption by at least 50%. Such approach has been adopted in other smoking cessation studies^28^,^29^.

## Results

### Baseline Study

Table 1 shows the baseline characteristics of the patients. Of 16,465 patients with DM who were screened, 890 (5.4%) were eligible and 557 (62.6%) consented. Consenting patients were randomly assigned to either the intervention (283 patients) or control group (274 patient). Most were male (88.3% male/11.7% female) and had attained secondary education or above (67.7%), and their mean age was 56 ± 11.4 years. Fewer patients in the intervention group (17.6%) reported having physician consultation in the past 30 days than in the control group (24.7%; p < 0.05). However, regression analysis showed that the higher rate of physician consultation did not predict smoking cessation at 6 and 12 months.

**Figure 1:**
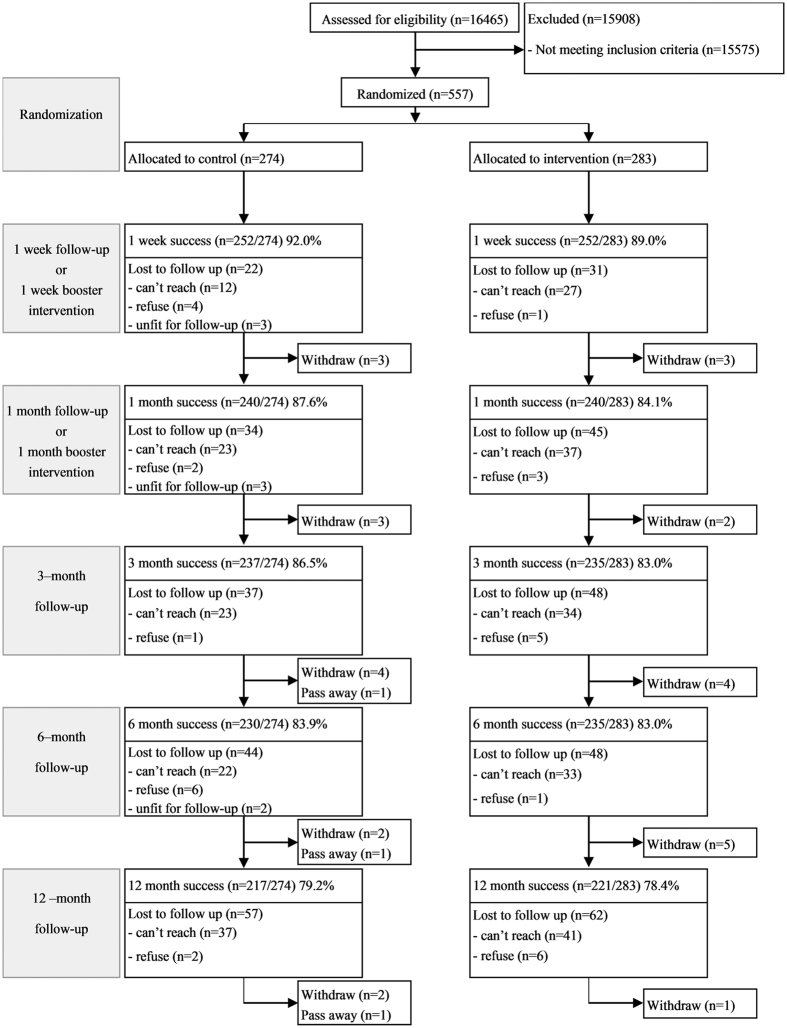
The Consolidated Standards of Reporting Trial flowchart to track participants through randomized controlled trial.

Other demographic characteristics including smoking, DM, and health status were similar between groups. Overall, participants smoked 14 cigarettes per day, and about half had moderate to severe nicotine dependence (Fagerstrom test score of 4 or higher). More than 70% were in the pre-contemplation stage of quitting. Although the mean duration since their diagnosis of DM was more than 10 years, about 80% of them perceived their health status as good. The 12-month retention rates for the experimental and control groups were 78.4% and 79.2%, respectively. A Consolidation Standards of Reporting Trials flowchart is shown in Fig. 1. Throughout the study, no adverse effects were reported.

### Interim analysis

In April 2014, 185 of 237 (78.1%) participants in the intervention group and 185 of 234 (79.1%) participants in the control group had been successfully evaluated at the 12-month follow-up. The smoking quit rate per protocol analysis for the intervention and control groups was 11.9% (22/185) and 17.3% (32/185), respectively, (rate ratio = 0.69, 95% CI 0.40–1.19, p = 0.17). By intention-to-treat analysis, the quit rate for the intervention and control groups were 9.4% and 13.7%, respectively. We stopped the recruitment early as the double triangular test showed that the abstinence rate with intention-to-treat analysis had achieved futility under the assumption that the 12-month quit rates of both groups was stable and further addition of subjects would not contribute to the significant changes in the results.

### Intervention outcomes

The effects of the brief, stage-matched smoking cessation counseling on the primary and secondary outcomes are shown in Table 2. No statistically significant difference was seen in the 7-day point-prevalence of smoking abstinence, the HbA1c level, the biochemically validated quit rate, the rate of smoking reduction by at least 50%, the stage of readiness to quit, or the number of quit attempts between the intervention and control groups at 12 months. The results of an independent t-test showed that regardless of the group allocation, there was no statistically significant difference in the HbA1c levels between quitters and non-quitters at 12 months (Table 3). The results of GEE also showed that there were no significant differences in the changes of HbA1c level among quitters at each time point, but there was a significant decreased trend for non-quitters with OR = 0.83 (95% CI: 0.71–0.97; *p* = 0.022) compared to baseline after adjusting to time, grouping of intervention and usual care, gender and age.

### Prediction factors in follow-up periods

Logistic regression showed that the intervention group had similar rates of abstinence at 12 months in both crude (OR, 0.63; 95% CI, 0.37–1.07) and adjusted (OR, 0.96; 95% CI, 0.48–1.92) models (Table 4). In the model adjusted for sociodemographic variables, quitting characteristics, and HbA1c level at baseline, smokers with greater daily cigarette consumption at baseline were less likely to quit smoking at 6 months (OR, 0.95; 95% CI, 0.91–0.99) and 12 months (OR, 0.93; 95% CI, 0.89–0.98) than those with lower daily cigarette consumption. Also, the smokers who attained secondary (OR, 0.40; 95% CI, 0.21–0.76) and post-secondary education (OR, 0.24; 95% CI, 0.07–0.87) were less likely to quit smoking at 12 months than those who attained a lower education level. Notably, we found that patients’ HbA1c levels at baseline could not predict their quitting at either 6 months (OR, 1.09; 95% CI, 0.93–1.27) or 12 months (OR, 0.92; 95% CI, 0.77–1.10).

## Discussion

The present study is the largest RCT to examine the effectiveness of an intervention on smoking cessation and glycemic control in smokers with DM and the first study to examine an intervention that consisted of brief stage-matched smoking cessation counseling in Chinese patients with type 2 DM^27^. Our RCT design followed the suggestions from previous studies of smoking cessation in adults with DM^7^,^23^. The design involved the incorporation of DM-specific intervention education about the increased health risk and assessment of patients’ smoking and smoking cessation status, including the HbA1c level after 1 year. The Bayes factor was 0.14 under the specified effect size in the power calculation (OR = 2), which supported the null hypothesis.

The overall results showed no significant differences in either the primary or the secondary outcomes between the intervention and control groups. There are some possible reasons for the non-significant results. Similar to a previous study of smoking behavior and perceptions among Hong Kong Chinese patients with type 2 DM^19^, our study showed that about 80% of patients perceived their health status as good, and thus might show reluctance to quit. This finding is further supported by the fact that more than 70% of the patients were in the pre-contemplation stage of quitting. These results agree with previous studies that found that smokers with DM, particularly those in the pre-contemplation stage, were less likely to quit and more likely to reject smoking cessation intervention^36^,^37^.

Another possible reason for the non-significant results might be the large proportion of chronic or even “hardcore” smokers in our study. Although the definition of “hardcore” smoker has not yet been well defined, six characteristics have been suggested, including (a) high daily cigarette consumption (15/day or more), (b) a high level of nicotine dependence (Heaviness of Smoking Index of 5/6), (c) daily smoking, (d) long-term smoking (5 years or more of regular smoking), (e) lack of intention to quit, and (f) lack of a lifetime quit attempt^56^. The patients in our study fulfilled three of these criteria (c, d, and e) and partially fulfilled one (a). They consumed an average of slightly fewer than 15 cigarettes daily, but which is still higher than in the general Hong Kong population (13 cigarettes). Most patients had attempted to quit (>65%) and might have encountered repeated failures in previous quitting attempts and were perhaps discouraged after these unsuccessful experiences.

Brief advice from a physician or health care professional was found to be effective in increasing the smoking cessation rate in the general population^57^,^58^. Our findings are consistent with the meta-analysis in patients with DM in the lack of efficacy of smoking cessation intervention involving brief counseling by doctors or nurses and complemented by follow-up and self-help materials (eight trials with 872 participants reviewed, with a risk ratio of 1.32; 95%CI, 0.23–7.43)^27^.

The brief (20-minute) counseling session in our study, involving stage-matched and risk communication might not be sufficiently intensive to trigger chronic or “hardcore” smokers to quit smoking as since the daily cigarette consumption could reach more than 10 for both groups. Counseling for pre-contemplators was probably too weak, especially for those who had many past failures. Several studies have reported that hard-core smokers are less likely to be affected by tobacco control interventions or policies^59^,^60^.

Another possible reason for the non-significant results might be the evidence for the causality of smoking in DM^61^, which was not established at the time of the intervention. We could only explain to the patients the association—not the causation—between DM and smoking. This might have undermined the patients’ willingness to quit smoking.

There is evidence that smoking is associated with increased levels of HbA1c in patients with DM^6^. Therefore, smoking cessation is hypothesized to lead to deceased HbA1c levels. The results of our study show that quitting smoking did not have any effect on HbA1c levels at the 12-month follow-up. One possible explanation lies in the difficulty of determining whether HbA1c levels could be expected to respond to smoking cessation after 12 months. The HbA1c levels might be less responsive or might require a longer time to respond to smoking cessation. Another study from Lycett and her colleagues from a cohort study (n = 10,692 adult type 2 diabetes smokers) argued that level of HbA1c increased temporarily by 0.21% at the first year of post-cessation time without association to weight change. The HbA1c level dropped when abstinence continued and became comparable to the current smokers three years later. Yet, the study did not take account for the dietary changes or exercise level of those quitters after smoking cessation, which may affect the rise of HbA1c level^62^. Indeed, previous studies showed that stopping smoking reduced the risk of DM^6^, but the beneficial effects were only apparent after 5 years of smoking cessation. Also, the risk of developing cardiovascular diseases would be decreased by half in quitters with diabetes suggested by another cohort study of 25 years follow up^63^.

We found that patients who smoked more cigarettes per day were less likely to quit smoking at 6 and 12 months, which is consistent with the findings of previous studies^64^. Because no association was found between past quit attempts and the quit rate at 6 and 12 months, daily cigarette consumption could be more useful for health care professionals to predict the quit rate of smokers with DM.

Our findings may not be generalizable to all patients with DM, particularly for those who smoked less than two cigarettes per day and thus were not included in this study. We encountered difficulties in subject recruitment due to an unexpectedly low prevalence of smoking in our DM patients. Also, the Independent Data Monitoring Committee recommended, an end to subject recruitment because the interim analysis showed the futility of further recruitment, and thus no benefit could be expected from the intervention. The difficulties in collecting HbA1c data resulted in missing data across the timeline, which might have contributed to the non-significant findings. Nevertheless, there was only 19.1% of missing values in the whole dataset, which is considered to be common and acceptable in psychological studies^65^.

Despite the non-significant results, the findings of this study have important implications for clinical practice and research. The most important implications for practice relate to the smoking determination of the Hong Kong Chinese patients with DM. Our study adds further evidence that smokers with DM in the pre-contemplation stage of quitting are less likely to quit and more likely to reject smoking cessation. Therefore, it is thus of paramount importance to design intensive and innovative interventions that use strong warnings and clearly communicate the risks (and extra risks) of continued smoking as a strategy to enhance patients’ motivation to quit. Furthermore, approaches to promote cessation that highlight the relevance and salience of health information by making it personally relevant (tailored) and address the specific characteristics of patients with DM (targeted) are needed^66^. In addition, as new evidence for the causality of smoking on DM becomes established, future trials should clearly explain the causal effects of smoking on DM.

Furthermore, because the benefits of quitting smoking, particularly the reduction in HbA1c levels, may require some time to respond to smoking cessation, it is recommended that future longitudinal studies be conducted to detect the benefit of giving up smoking over an extended period in patients with DM.

The results show that a brief intervention is not associated with a decrease in smoking habits in DM patients. These patients are less likely to quit and more likely to reject smoking cessation intervention. More comprehensive and innovative interventions that use strong warnings and communicate clearly the risk of continued smoking as a strategy to enhance the motivation of smokers with DM to quit, should be tested and evaluated in future studies.

## Additional Information

**How to cite this article**: Li, W. H. C. *et al*. Brief intervention to promote smoking cessation and improve glycemic control in smokers with type 2 diabetes: a randomized controlled trial. *Sci. Rep.* **7**, 45902; doi: 10.1038/srep45902 (2017).

**Publisher's note:** Springer Nature remains neutral with regard to jurisdictional claims in published maps and institutional affiliations.

## Figures and Tables

**Table 1:** Baseline characteristics of the patients (N = 557).

	Intervention	Control
	n = 283	n = 274
Demographic		
Age, mean ± SD, years	56.0 ± 11.3	55.5 ± 11.5
Male sex, n *(%)*	247 (87.3)	245 (89.4)
Level of education, n (%)		
Primary or below	84 (29.7)	85 (31.0)
Secondary	170 (60.1)	156 (56.9)
Post-secondary	24 (8.5)	27 (9.9)
Missing	5 (1.7)	6 (2.2)
Employment status, n (%)		
Housewife	5 (1.8)	10 (3.6)
Full time student	1 (0.4)	1 (0.4)
Retired	83 (29.3)	74 (27.0)
Unemployed	21 (7.4)	23 (8.4)
Employed	173 (61.1)	166 (60.6)
Smoking history		
No. of years of regular smoking (≥1 cigarette per day), mean ± SD	38.1 ± 12.8	37.8 ± 12.9
Daily cigarette consumption, mean ± SD	14.2 ± 9.0	13.5 ± 9.2
Fagerstrom test score for nicotine dependency, n (%)		
≤3 (mild)	142 (50.2)	141 (51.5)
4–5 (moderate)	85 (30.0)	93 (33.9)
≥6 (severe)	50 (17.7)	40 (14.6)
Missing	6 (2.1)	0 (0)
Past quit attempt (quit for at least 24 hours), n (%)		
Yes	176 (62.2)	191 (69.7)
No	107 (37.8)	83 (30.3)
Stage of readiness to quit, n (%)		
Pre-contemplation	215 (76.0)	200 (73.0)
Contemplation	46 (16.3)	53 (19.3)
Preparation	22 (7.8)	21 (7.7)
Self-efficacy score^a^ Mean ± SD	33.0 ± 8.5	33.3 ± 8.0
DM history		
No. of years of being diagnosed with DM, mean ± SD	10.6 ± 8.3	10.2 ± 8.2
DM complication^b^ n (%)		
Yes	159 (56.2)	170 (62.3)
No	118 (41.7)	103 (37.7)
Missing	6 (2.1)	0 (0)
Health		
Hospitalized in the past 6 months, n (%)	51 (18.5)	46 (16.9)
Doctor consultation in the past 30 days, n (%)	48 (17.6)	67 (24.7)
Self-rated health, n (%)		
Very Bad	3 (1.1)	1 (0.4)
Bad	47 (16.6)	53 (19.3)
Good	209 (73.9)	199 (72.3)
Very good	1 (0.4)	2 (0.7)
Missing	23 (8.0)	19 (7.3)
Other co-morbidities, n (%)		
Hypertension	139 (49.1)	148 (54.0)
Cardiovascular diseases	33 (11.7)	32 (11.7)
Respiratory diseases	12 (4.2)	14 (5.1)
Digestive diseases	13 (4.6)	14 (5.1)
Blood Profile, mean ± SD		
HbA1c level (%)	8.1 (1.7)	8.2 (1.7)
Low-density lipoprotein (LDL) (mmol/L)	2.6 ± 0.8	2.6 ± 0.8
High-density lipoproteins (HDL) (mmol/L)	1.1 ± 0.4	1.1 ± 0.4

Note. ^a^Score ranged from 12–60, the higher the score, the more self-efficacy the person; ^b^DM complications refer to cardiovascular disease, stroke, neuropathy, eye complications, nephropathy (kidney disease), and other DM related complications.

**Table 2:** Quit rate, smoking reduction rates and quit attempts in intervention and control groups^a^.

	Intervention group (n = 283)	Control group (n = 274)	Effect Size	p-value
**Primary outcome at 12 months**				
Self-reported 7-day quit rate	26 (9.2)	38 (13.9)	0.073	0.08
**Secondary outcome**				
Biochemically validated quit rate, n (%)				
12 months	9 (3.2)	14 (5.1)	0.049	0.25
Self-reported reduction rate^b^ n (%)				
3 months	29 (10.2)	46 (16.8)	0.096	0.02
6 months	38 (13.4)	39 (14.2)	0.012	0.78
12 months	42 (14.8)	40 (14.6)	0.003	0.94
Blood Profile				
HbA1c level at 12 months^c^ mean ± SE				
%	7.95 ± 0.11	8.05 ± 0.11	−0.41	0.49
mmol/mol	63 ± 1.2	64 ± 1.2	−0.38	0.49
At action stage of readiness to quit, n (%)				
3 months	22 (7.8)	17 (6.2)	0.031	0.47
6 months	32 (11.3)	33 (12.0)	0.012	0.89
12 months	33 (11.7)	47 (17.2)	0.078	0.12
Had quit attempt(s) for at least 24 hours since last assessment, n (%)				
3 months	41 (14.5)	44 (16.1)	0.022	0.61
6 months	45 (15.9)	50 (18.2)	0.031	0.40
12 months	45 (15.9)	57 (20.8)	0.063	0.14

Note. ^a^By intention-to-treat analysis, assumed all non-responded follow-up patients as current smoker, not at action stage of readiness to quit and no quit attempt in past 24 hours; and ^b^Reduction by at least 50% from baseline; and ^c^Adjusted for anti-diabetic medication step-up adjustment at 6-month.

**Table 3:** Comparison of level of HbA1c (%) and changes from baseline to 12-month between 12-month quitters and non-quitters (N = 557).

	Quitters (n = 64)	Non-quitters (n = 493)	p-value
Mean HbA1c (%)^a^			
12-month	7.96	7.99	0.90
Baseline	7.94	8.17	0.32
HbA1c level changes (95% CI)^b^			
12-month Baseline	1.02 (0.75–1.40) 1	0.83 (0.71–0.97)* 1	/

Note. ^a^Independent sample t-test ^b^Generalized Estimating Equation (GEE) analysis adjusted by time, grouping of intervention and usual care, gender and age.

*p < 0.05.

**Table 4:** Factors predicting smoking cessation at 6 and 12 months follow-up.

Variables	Odds Ratio (95% Confidence Interval)	
	6-month (N = 557)	12-month (N = 557)
**Unadjusted model**		
Control group	1.00	1.00
Intervention group	1.00 (0.59–1.69)	0.63 (0.37–1.07)
**Adjusted model**^a^		
Study group		
Control group	1.00	1.00
Intervention group	0.97 (0.55–1.68)	0.68 (0.69–1.21)
Sex		
Male	1.00	1.00
Female	1.4 (0.65–3.03)	1.5 (0.70–3.20)
Age	0.99 (0.97–1.02)	0.99 (0.97–1.02)
Education		
Primary or below	1.00	1.00
Secondary	0.54 (0.28–1.05)	0.40 (0.21–0.76)
Post-secondary	0.44 (0.14–1.39)	0.24 (0.07–0.87)
Baseline daily cigarette consumption	0.95 (0.91–0.99)	0.93 (0.89–0.98)
Baseline past quitting attempt		
No	1.00	1.00
Yes	1.49 (0.80–2.80)	1.66 (0.88–3.13)
Baseline HbA1c level	1.09 (0.93–1.27)	0.92 (0.77–1.10)

Note. ^a^Model adjusted for all the variables listed.
